# Long-lasting complete remission in a patient with systemic metastases of recurrent breast cancer treated with cyclin-dependent kinases 4/6 inhibitors: a case report

**DOI:** 10.1186/s13256-023-03902-4

**Published:** 2023-05-09

**Authors:** Toshihiko Yoneto, Kenichiro Hasumi, Nobukazu Takahashi, Nastuki Seki, Yasutaka Takeda, Takayuki Yoshimoto

**Affiliations:** 1 Department of Breast Surgery, Hijirigaoka Hospital, Tama City, 2-69-6 Renkōji, Tokyo, 206-0021 Japan; 2 Department of Breast Surgery and Oncology, Nippon Medical School, Chiba Hokusou Hospital, 1715 Kamagari, Inzai City, Chiba, 270-1694 Japan; 3 Department of Immunoregulation, Institute of Medical Science, Tokyo Medical University, 6-1-1 Shinjuku, Shinjuku-Ku, Tokyo, 160-8402 Japan; 4 Department of Surgery, Oguchihigashi General Hospital, 2-19-1 Irie, Kanagawa-Ku, Yokohama, Kanagawa 221-0014 Japan; 5 Breast Oncology Center, Fukujuji Hospital, 3-1-24 Matsuyama, Kiyose, Tokyo 204-8522 Japan

**Keywords:** Breast cancer, CDK4/6 inhibitors, Complete remission, Immune systems, Immunosenescent T cell, Recurrence

## Abstract

**Background:**

The prognosis for recurrence cases of hormone receptor-positive HER2-negative breast cancer remains poor, and treatment strategies that emphasize quality of life have often been chosen, with few physicians aiming for a cure. Our objective is to assess the validity of such current treatment strategies.

**Case presentation:**

A 74-year-old Asian woman with multiple lung and liver metastases after local recurrence of breast cancer was treated with two different cyclin-dependent kinases 4/6 inhibitors sequentially in combination with endocrine therapy. Flow cytometric analysis of the patient’s peripheral blood mononuclear cells was also performed to evaluate the host’s immune status. Complete remission was achieved without cytotoxic agents and the patient remains disease free to this day, 6 years after the initial relapse. Additionally, no increase in the population of the immunosenescent T cells with a phenotype of CD8^+^CD28^−^ was observed in the patient’s peripheral blood mononuclear cells, suggesting that the immune system was well maintained.

**Conclusions:**

We present this case study to develop new treatment strategies for recurrent breast cancer that is not only bound to misinterpretations of the Hortobagyi algorithm, but also aim for a cure with noncytotoxic agents to maintain the host’s immune system and early detection of recurrence.

## Introduction

Currently, combination therapy with cyclin-dependent kinase (CDK) 4/6 inhibitors and endocrine therapy for hormone receptor-positive (HR^+^) HER2-negative (HER2^−^) recurrent breast cancer is generally chosen as a treatment strategy that emphasizes quality of life without aiming for a cure. Indeed, there are few reports of complete remission (CR) being achieved and subsequently maintained in the long term. Agents with novel mechanisms, such as CDK4/6 inhibitors, have antitumor effects comparable to those of cytotoxic agents and are less likely to impair the host’s immune system. Furthermore, abemaciclib has a slightly different pharmacological structure to palbociclib and ribociclib, and has antitumor effects related to the host’s immune system other than cell cycle arrest. With the rapid development of these new agents, alternative treatment strategies for recurrent breast cancer may be required, including the option of aiming for a cure.

We experienced a unique case of postmenopausal HR^+^HER2^−^ breast cancer with multiple lung and liver metastases after local recurrence, which achieved CR by switching from palbociclib to abemaciclib as a CDK4/6 inhibitor, and subsequently remained disease free. Additionally, we examined the immunosenescent T cells with a CD8^+^CD28^−^ phenotype of the patient’s peripheral blood mononuclear cells (PBMC) by the flow cytometric analysis to further elucidate the role of the host’s immune status. On the basis of this experience, our goal is to define the direction of new treatment strategies to achieve a cure for recurrent breast cancer that also focuses on the host’s immune system and the selection criteria for candidate patients by deconstructing conventional treatment strategies.

## Case presentation

A 74-year-old Asian woman presented to our hospital with a lump in her right breast, for which partial mastectomy and axillary lymph node dissection (ALND) were performed. No medical or family history for the patient was noted. Pathological analysis showed invasive ductal carcinoma, solid-tubular type, pT2N1aM0 with stage IIB. Immunostaining results were 90% estrogen receptor (ER)-positive, 5% progesterone receptor (PgR)-positive, and 5% Ki-67-positive, but HER2-negative expression. The patient refused both chemotherapy and radiotherapy as postoperative treatment and so only an aromatase inhibitor was administered.

Two years and one month after the first operation, computed tomography (CT) imaging showed an irregular mass in the pectoralis major muscle layer and enlarged axillary lymph nodes. On the basis of the diagnosis of local recurrence, total mastectomy combined with pectoralis major muscle resection and ALND was performed 2 years and 3 months later. Pathological analysis showed recurrent invasive ductal carcinoma and more than five metastatic axillary lymph nodes, HER2^−^, 90% ER, 5% PgR, and 20–30% Ki-67 index. The patient again refused the chemotherapy and was therefore only treated with postoperative radiotherapy (50 Gy in 25 fractions).

**Fig. 1:**
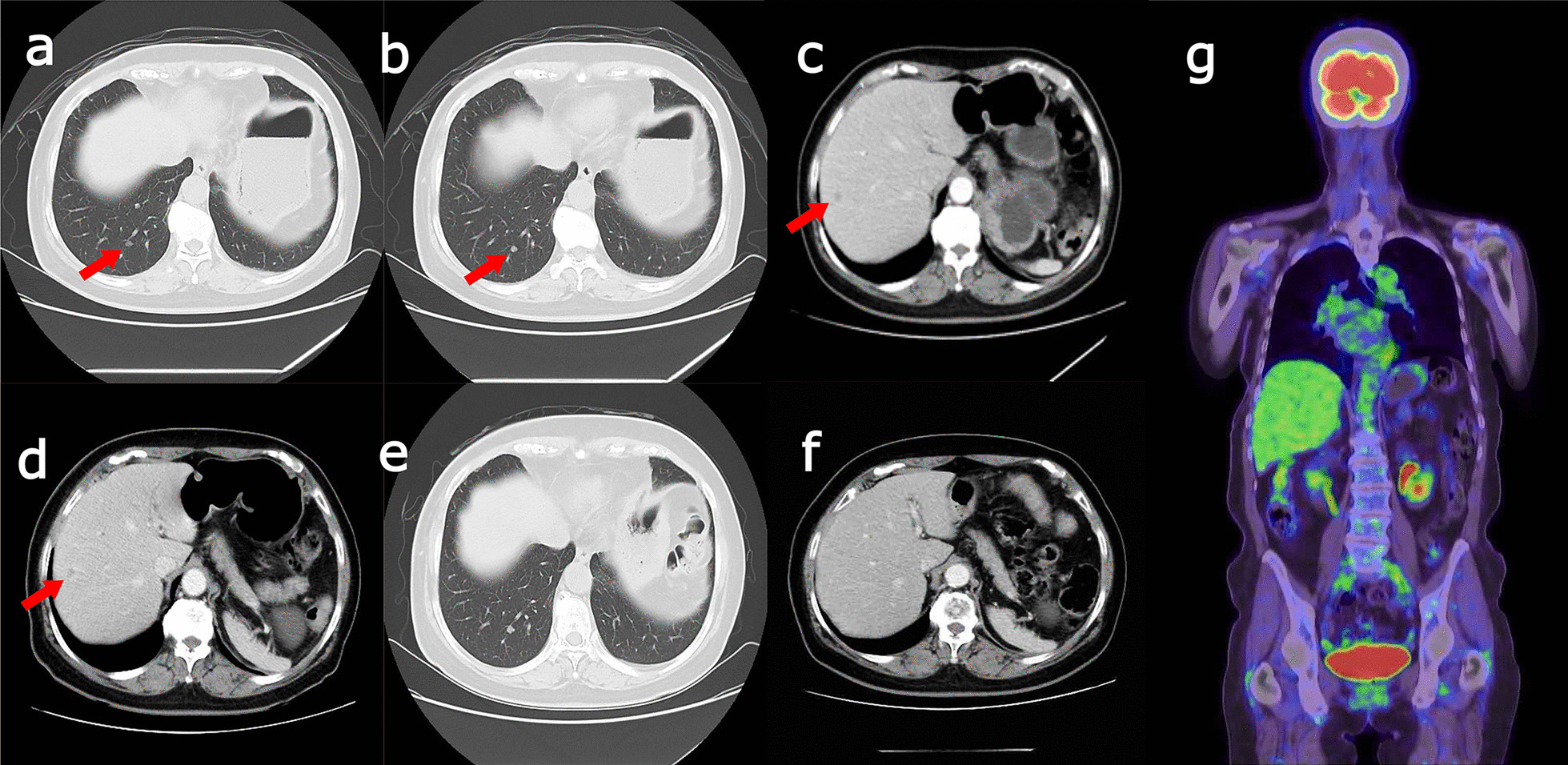
Images at the time of the patient’s second recurrence. Computed tomography showed two metastases to the inferior lobe of the right lung (**a**: arrows) and a small metastatic lesion in the liver (**b**: arrows). The liver metastasis had slightly increased 7 months after the second recurrence (**c**: arrows). At 4 months after the start of palbociclib, the liver metastases had receded slightly (**d**: arrows). At 7 months after the start of palbociclib, the lung metastasis had disappeared completely (**e**). At 1 year and 5 months after the start of abemaciclib, the liver metastasis had completely disappeared (**f**). Integrated positron emission tomography–computed tomography showed no metastasis or recurrence in any organ, including the lungs and liver (**g**)

Two years and ten months after the first operation, CT imaging showed two metastases in the right lung (Fig. 1a, b) and a small metastatic lesion in liver segment VII/III (Fig. 1c). We recommended changing the currently used aromatase inhibitor, and a fulvestrant was initiated. After the liver metastasis worsened slightly (Fig. 1d), palbociclib was added starting 3 years and 8 months later, and 4 years and 3 months later, the lung metastases completely disappeared (Fig. 1e). Although strong myelosuppression emerged as an adverse event, forcing a change from palbociclib to abemaciclib 4 years and 8 months later, even the liver metastases had completely disappeared by 6 years and 1 month later (Fig. 1f). Integrated positron emission tomography (PET)–CT performed 6 years and 3 months later revealed no sign of metastasis or recurrence in any organs. (Fig. 1g).

**Fig. 2:**
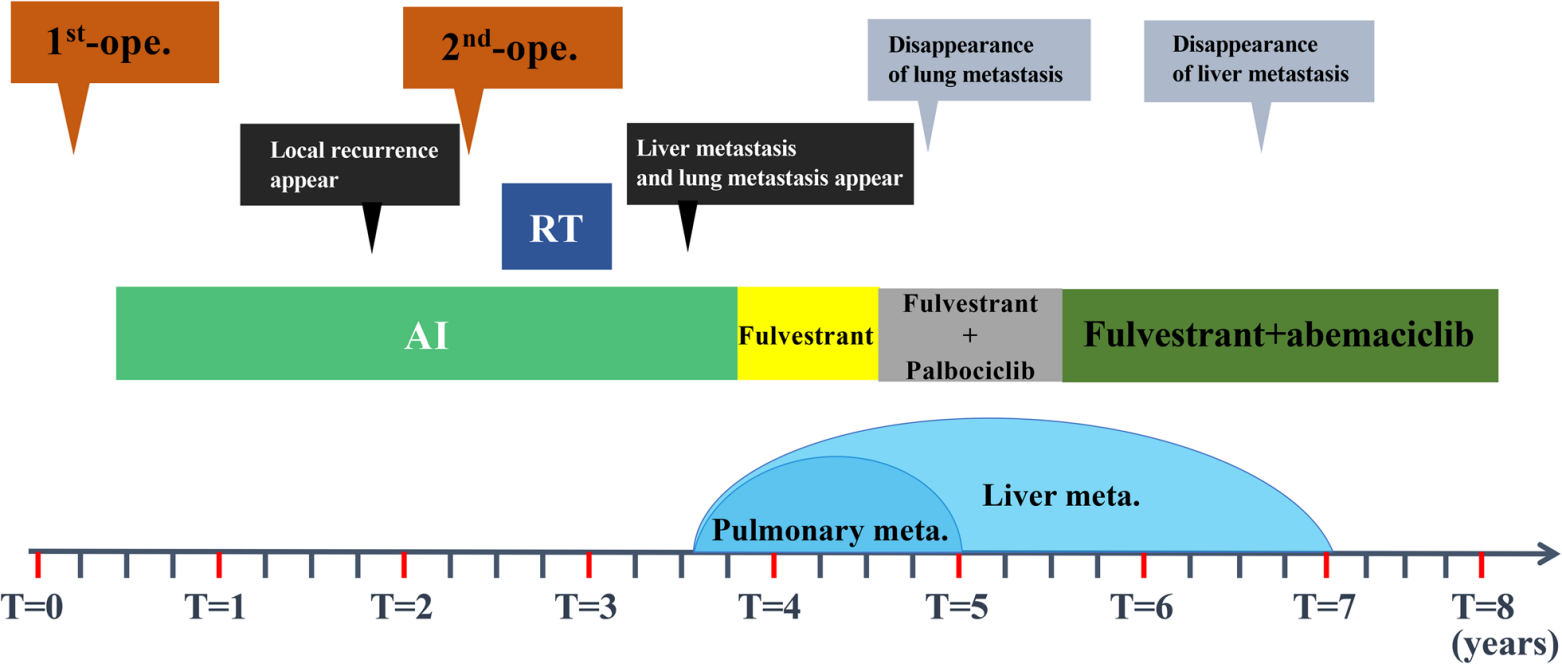
The patient’s entire clinical course from the time of admission to the time of writing

Figure 2 charts the patient’s clinical course from admission to the time of writing.

**Fig. 3:**
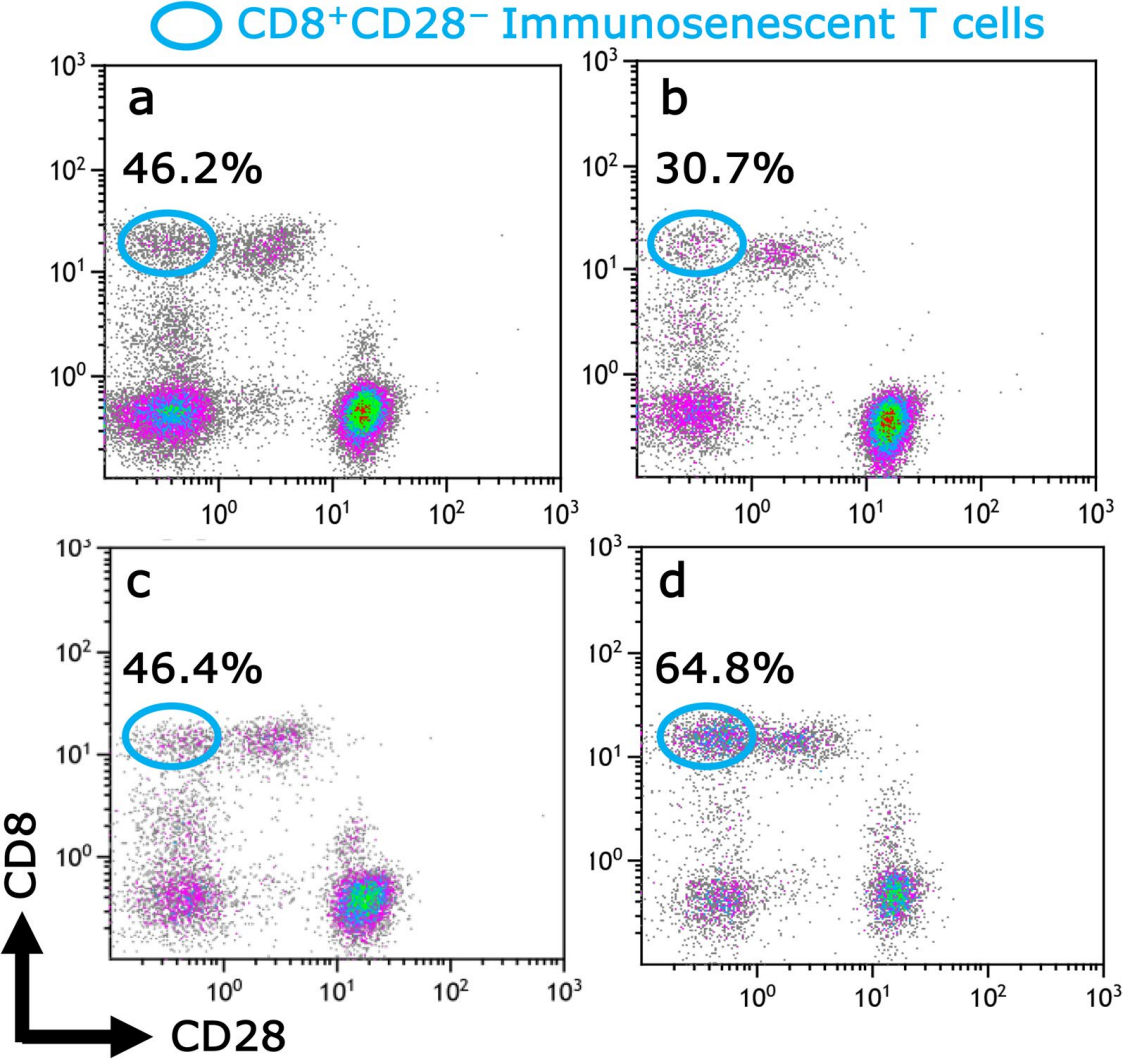
Analysis by flow cytometry of the peripheral blood mononuclear cells showed that the population of immunosenescent T cells with a phenotype CD8^+^CD28^−^ was 46.2% in this patient (**a**), 30.7% in another 72-year-old patient with breast cancer with bone metastases who had no previous treatment with and was currently being treated with abemaciclib (**b**), 46.4% in a healthy volunteer (**c**), and 64.8% in a patient with recurrent breast cancer treated with multiple cytotoxic agents (**d**). Blue circles indicated the population of immunosenescent T cells (CD8^+^CD28^−^)

Six years and nine months after the first operation, an analysis by flow cytometry of the patient’s PBMC was also performed. We confirmed that the population of immunosenescent T cells with a phenotype CD8^+^CD28^−^ (CD8^+^ T_sen_) was 46.2% (Fig. 3a) and not increased compared with a 67-year-old healthy volunteer (Fig. 3c). CD8^+^ T_sen_ populations were also not increased (30.7%) in another 72-year-old patient with breast cancer with bone metastases who had no previous treatment with cytotoxic agents and is currently treated with abemaciclib (Fig. 3b). In contrast, an elevated level of CD8^+^ T_sen_ to 64.8% was observed in a 66-year-old patient with breast cancer with solitary lung metastasis who had previously been administrated with multiple cytotoxic agents and was currently treated with abemaciclib (Fig. 1d). No differences in the proportions of natural killer (NK) and regulatory T (Treg) cells were observed among these patients (data not shown).

As of 8 years and 1 month after the first operation and 6 years after the first recurrence, no signs of cancer relapse have been detected and the patient continues to be treated with fulvestrant and abemaciclib with performance status of 0.

## Discussion

CDK 4/6 inhibitors combined with endocrine therapy are an effective treatment strategy for HR^+^HER2^−^ recurrent breast cancer, but the CR rate is low in clinical trials, such as in the PALOMA-3 trial, which reported 0% CR and 10.4% partial response [1]. Therefore, these results also could not extensively change the treatment strategies on the basis of the misinterpreted Hortobagyi algorithm, which prolongs life while maintaining good quality of life without aiming for a cure, even considering subgroup analyses of ESR1 and PIK3CA mutations. However, these results are not synonymous with the impossibility of treatment strategies aimed at curing recurrent breast cancer with novel agents possessing novel mechanisms, such as CDK4/6 inhibitors. For this to be possible, we should not be bound by conventional preconceptions and should develop new strategies using these weapons.

The first concern with these new strategies relates to the sequential and modified administration of CDK4/6 inhibitors. In the case reported here, the patient has been treated with two different CDK 4/6 inhibitors, and the first-line treatment with palbociclib resulted in a partial response. Subsequently, the side effects forced a change from palbociclib to abemaciclib, but a CR was eventually achieved. The sequential changes of CDK4/6 inhibitors for treating recurrent breast cancer have yet to prove efficacy [2]. Recent studies have reported that abemaciclib-induced cross-resistance to other CDK4/6 inhibitors *in vitro*, but another report of post-marketing surveillance data showed that cross-resistance did not exist. Therefore, conclusions regarding the presence or absence of cross-resistance between CDK4/6 inhibitors require substantial future research. Nonetheless, we have concluded that it may be well worth considering switching from other CDK4/6 inhibitors to abemaciclib. The reason is that abemaciclib has an antitumor effect not only through cell cycle arrest, but also through the induction of cellular senescence, apoptosis, and suppression of Treg cells [3]. Due to these effects, abemaciclib has been approved by the U.S. Food and Drug Administration (FDA) for monotherapy without endocrine therapy. In fact, 3.5% of subjects achieved CR in the MONARCH2 trial [4], and more CR may be achieved in practice as the increased opportunities for both the use of CDK4/6 inhibitors and the sequential changes in the future.

Concurrently, the treatment strategies for recurrent breast cancer in subtypes other than HR^+^HER2^−^ are also important issues that need to be mentioned. CDK4/6 inhibitors combined with anti-HER2 agents, immune checkpoint inhibitors, and nuclear signaling inhibitors could be effective, and many clinical trials are currently underway. The monarchHER trial suggested that CDK4/6 inhibitors act through crosstalk in signaling pathways downstream of tyrosine kinase receptors such as HER2 [5]. Furthermore, CDK4/6 inhibitors have been reported to arrest the cell cycle via a hormone-receptor-independent mechanism in the luminal androgen receptor type of triple-negative breast cancer [6]. Additionally, as mentioned above, abemaciclib has been reported to be involved in the host’s immune system through varied mechanisms. This means that synergistic effects could be expected from the combination of abemaciclib with immune-related agents such as anti-PD-L1, CTLA-4 inhibitors, and antibody therapy against Treg cells, not only in HR^+^HER2^−^ subtype, but also in various subtypes [7].

The second concern of these relates to the importance of detecting early recurrence. Although it may seem obvious, early detection is crucial to cure recurrent breast cancer. The reason for this is that early detection of the recurrence, while the absolute number of tumor cells is minimal and does not affect the host’s immune system, may enable curing with only noncytotoxic agents. Therefore, we consider that intensive postoperative follow-up must be performed to achieve a cure in patients at high risk of recurrence, despite various opinions [8].

The third concern of these relates to the host’s immune system. Although there has been scant attention to the host’s immune system, it is undoubtedly one of the most important components in the treatment strategies for recurrent breast cancer, playing a twin role with the information of the tumor cells themselves. Therefore, idealistically speaking, we should predict the absolute number of tumor cells by liquid biopsy at the time of recurrence detection, while the state of the host’s immune system should be confirmed by immunophenotyping of the PBMC or their tumor-infiltrating lymphocytes (TILs). If the host’s immune status may be well maintained and tumor cells are scarce, the treatment strategies aiming for a cure with CDK4/6 inhibitors or immune-related agents [9], rather than cytotoxic agents, should be considered an option. Furthermore, such a treatment strategy may maintain the immune response system for a long time and increase the likelihood of a cure, even if subsequent multiple relapses occur.

The immunosenescent T cells with a CD8^+^CD28^−^ phenotype (CD8^+^ T_sen_) have generally been accepted to be immunosuppressive and increase in the population of those as the immune system is attenuated [10]. No increased population of CD8^+^ T_sen_ suggested that the host’s immune system was not impaired in the cases treated with noncytotoxic agents, which may have led to the long-term maintenance of a CR, considered a cure in this case. In this context, antitumor agents are essentially a “*pharmakon*” as a double-edged sword, but the agents with novel mechanisms may be interpreted as being more medicinal than poisonous.

On the basis of the above, it is our firm belief that early detection of recurrence through intensive postoperative follow-up, the analysis of the host’s immune status at the event of recurrence, and the treatment strategies with agents that cause as little damage as possible to the immune system may enable curing even if relapses occur. More fortunately, these treatment strategies might be theoretically effective not only in HR^+^HER2^−^ subtype, but also in all other subtypes. Therefore, therapy to aim for a complete cure using agents other than cytotoxic agents should be considered in the future. This is a single incident in which a patient refused to use the cytotoxic agents with strong side effects and had been treated with a CDK4/6 inhibitor, which coincidentally resulted in CR. Although we do not believe that such a treatment strategy is suitable for all patients, more studies are needed to determine the efficacy of such treatment in these cases.

We, as physicians, are sincerely looking forward to the day when we will not have to do the hard work of admonishing our patients to give up a cure once a relapse has been detected. Therefore, we should do our best to create a paradigm shift in treatment strategies for recurrent breast cancer that also emphasizes the maintenance of the host’s immune status.

## Conclusions

We report a patient with recurrent breast cancer with multiple pulmonary and hepatic metastases treated with two different CDK4/6 inhibitors, who achieved a CR and is disease free to this day, 6 years after the first recurrence. In the future, alternative treatment strategies with various agents possessing new mechanisms that do not impair immune status may deconstruct the dichotomy of the attacking tumor cells and the maintaining of the immune system, leading to a cure for recurrent breast cancer.

## Data Availability

All data generated or analyzed for this article are included in the article.

## Abbreviations

CD8^+^ T_sen_: Immunosenescent T cells
CDK4/6: Cyclin-dependent kinase 4/6
CR: Complete remission
CT: Computed tomography
HER2: Human epidermal growth factor receptor type 2
HR: Hormone receptor
PBMC: Peripheral blood mononuclear cell
